# Acceptability of Active and Passive Data Collection Methods for Mobile Health Research: Cross-Sectional Survey of an Online Adult Sample in the United States

**DOI:** 10.2196/64082

**Published:** 2025-09-12

**Authors:** Nelson Roque, John Felt

**Affiliations:** 1 Center for Healthy Aging, The Pennsylvania State University, 422 Biobehavioral Health Building, University Park, PA, 16801, United States, 1 814-863-7502; 2 Department of Psychology, University of Central Florida, Orlando, FL, 32816, United States

**Keywords:** mHealth, privacy, smartphones, sensors, acceptability, mobile health, attitudes, opinions, digital health technologies, online survey, surveys, data privacy, questionnaires

## Abstract

**Background:**

Digital health technologies, including wearable devices and app-based cognitive and health assessments, are pervasive and crucial to better understanding important public health problems (eg, Alzheimer’s disease and related dementias). Central to understanding mechanisms driving individuals’ willingness to share various data streams are concerns regarding data privacy, security, and control over generated data.

**Objective:**

This survey was designed to learn more about attitudes and opinions related to digital health technologies and the sharing of associated data.

**Methods:**

A total of 1509 adults were recruited from Prolific to complete an online survey via Qualtrics. Of these, 1489 participants provided valid data for analyses. Participants completed a structured survey consisting of multiple modules after informed consent was provided. These included: (1) demographic characteristics; (2) prior research experience; (3) mobility factors (eg, use of mobility aids, driving frequency); (4) technology ownership (eg, smartphones, tablets, home Wi-Fi); (5) social media use (eg, frequency of engagement with platforms such as Facebook, Instagram, and TikTok); (6) willingness to contribute different types of data across categories, including activities, sensors, and metadata; (7) opinions about data control and privacy options (eg, data deletion, stream-specific control); and (8) willingness to interact with assistive technologies such as robots, for Instrumental Activities of Daily Living.

**Results:**

The final cohort (N=1489) had a mean age of 35.5 years (SD 12.0), was 44% female (n=652), and predominantly identified as White (76%, n=1134), with high rates of smartphone ownership (99%, n=1479) and home Wi-Fi access (98%, n=1464). Participants were most willing to share data streams with clear health implications and least willing to share data streams with greater privacy or reidentification potential (eg, GPS location, in-vehicle dashcam footage). On average, people were willing to complete ambulatory cognitive assessments for 56.7 (SD 36.2) days, air quality monitoring for 58.1 (SD 37.7) days, and GPS location monitoring for 37 (SD 39.0) days. People expected control over their data, including the ability to delete all or specific streams of the data contributed for research. Most participants prioritized control over their data, with 71% (n=1061) favoring the ability to delete all data contributed for research purposes. Stream-specific data deletion (65%, n=960) and time-specific deletion (44%, n=653) were also valued; interest in sharing data with insurance providers (30%, n=453) or caregivers (26%, n=384) was notably lower.

**Conclusions:**

Findings have implications for the design of digital health technologies and education-related to the use and implications of collected data.

## Introduction

### Background

Digital health technologies, including app-based cognitive and psychosocial assessments and wearable sensor-based health monitoring devices, are pervasive and crucial for better understanding and predicting trajectories related to important public health outcomes such as Alzheimer’s disease and related dementias. The availability of detailed sensor data streams and data available via application programming interfaces creates new opportunities for scientific discovery, especially considering that passively collected data may be more robust for measuring aspects of daily life that can be influenced by observation, memory interference, or retrospective biases. The increase in easily shareable metadata (eg, Google Takeout) also creates a new fruitful avenue for research, however the extent public awareness and willingness to share these data and from other data streams remains unclear. Past studies have shown that in general, the overall acceptance for various smartphone-based tasks and sensing modalities is low, with variations in type of activity and sensor [1, 2]. However, repeated prompting for specific data streams has been shown to increase willingness to share [3]. In general, participants are interested in learning from their own data, and a proportion of these are also interested in sharing their information with a health care provider [4].

### Privacy, Security, and Data Controls

Critical to understanding mechanisms driving willingness to share various data streams are concerns about data privacy, security, and control over generated data [3, 5]Once the data are collected, different members of a research team, often across different institutions, work with deidentified versions of the datasets. In some cases, data are stored indefinitely for algorithm development—seeking new purposes or uses for the data that were not originally considered. In other cases, advances in machine learning have made it possible to reidentify individuals with enough precision to warrant security concerns over warehousing sensitive data without appropriate deidentification procedures [6]). As such, it is imperative to consider the most important stakeholder, the respondent and to assess their expectations regarding the data they generate for research and the level of controls they expect.

### Assessment: Active vs Passive?

**Figure 1.**
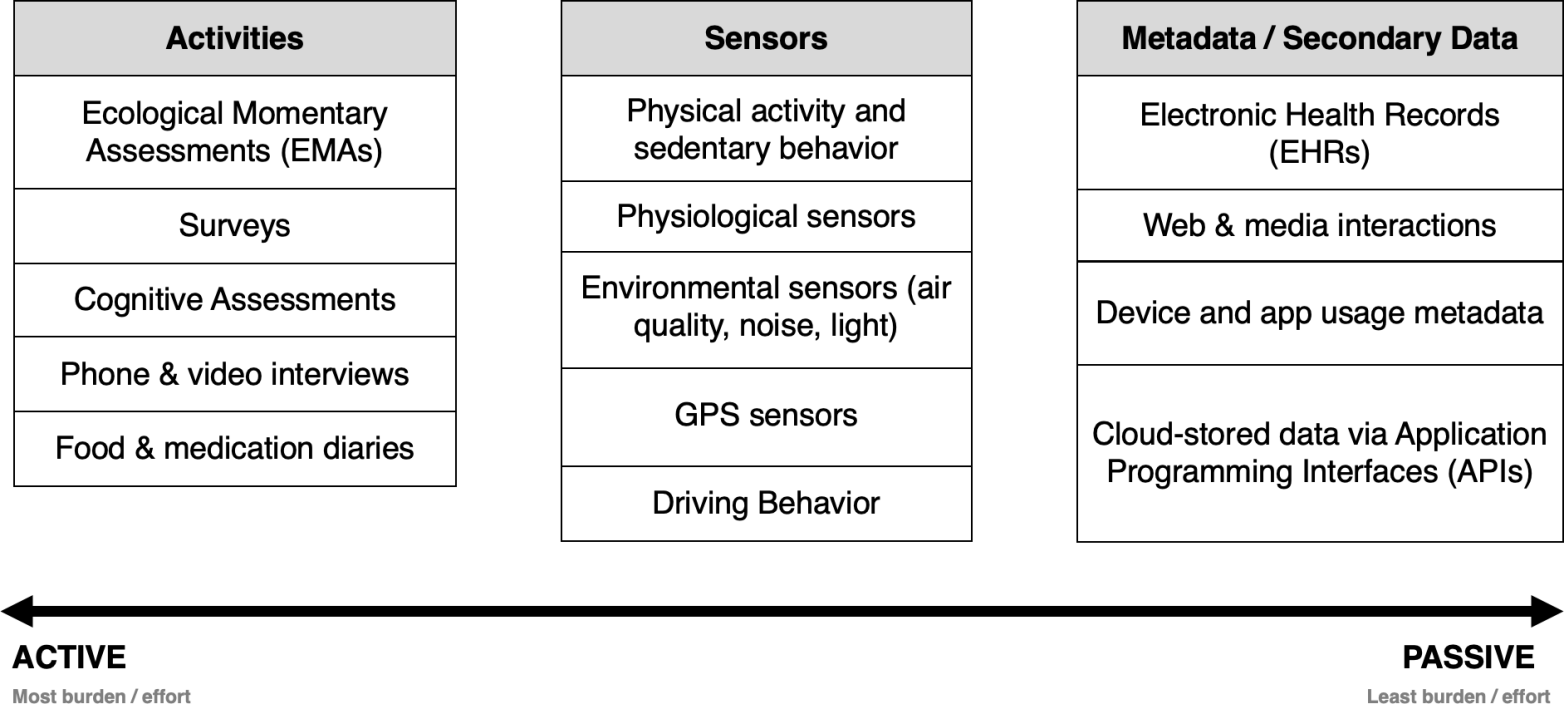
Active-passive continuum of research data streams: activities, sensors, and metadata (examples listed for each data stream category).

To answer important scientific questions, researchers are interested in collecting data across the spectrum of participant involvement and burden, ranging from active data collection activities (eg, completing a survey, cognitive assessments), to passive data collection (eg, extracting sensor data streams, reading existing metadata [7]; Figure 1). Passive data collection methods refer to data captured automatically without participant effort (eg, sensors, metadata), whereas active methods require participant involvement (eg, completing surveys or assessments). Active data collection also requires input from the respondent in a manner that makes the goal of the research more apparent. On the other hand, passive assessment does not requiring respondent burden or input, but rather, relies on data that is collected continuously in the background.

In general, the mechanisms driving willingness to contribute various data, irrespective of specific study demands, sponsors, and incentives [5] remain unclear. From the general patterns of attrition observed in longitudinal studies, it is apparent that a subset of participants may have a multitude of reasons for choosing to not engage in certain research activities.

### This Study

The purpose of this study is to investigate the acceptability of active and passive data collection methods in mobile health research. This survey was designed to explore attitudes and opinions related to digital health technologies and willingness to share related data. The three research questions guiding this work are:

1. RQ1: What types of data are people willing to contribute?
2. RQ2: For how long are people willing to contribute critical data streams?
3. RQ3: What type of data control and feedback do people expect?

## Methods

### Participants

A total of 1509 adults were recruited from Prolific [8]. The Prolific platform allows researchers to post a ‘task’ with a short description for potential participants to review before beginning. In addition to the study background language, we the potential participants were informed: “This survey is designed to learn more about your attitudes and opinions related to digital health technologies and related data/data-sharing.” Two participants did not consent to participate, and 18 did not complete the survey and were dropped from the study, resulting in a total of 1489 participants with a mean age of 35.5 (SD 11.97) years.

### Ethical Considerations

This work was carried out in accordance with The Code of Ethics of the World Medical Association (Declaration of Helsinki). The Institutional Review Board (IRB) at the University of Central Florida approved the study protocol described in this manuscript (IRB No: STUDY00002281). The survey was pilot tested by members of the research laboratory conducting the study, for issues of usability and technical functioning. The IRB determined this study as exempt from regulation for human subjects research. Participants were provided an opportunity to accept or refuse consent before beginning this voluntary survey. The consent form indicated the expected time commitment, compensation, and nature of study. If consent was refused, the survey would close for that participant. Participants received US $2 after completing the survey on the Prolific Research Platform. All data was deidentified; the authors are accountable for all aspects of the work (including full data access, integrity of the data and the accuracy of the data analysis) in ensuring that questions related to the accuracy or integrity of any part of the work are appropriately investigated and resolved.

### Materials and Design

Participants completed a battery of questionnaires via the Qualtrics survey platform, designed to assess sample demographic characteristics (eg, technology ownership, mobility), opinions about data control, and willingness to contribute various data for research. These modules were designed to assess various facets of attitudes and behavior relevant to longitudinal observational research, to determine heterogeneity in willingness to use devices or share data.

### Procedures

The participants completed a questionnaire via the Qualtrics survey platform, where the first page displayed the consent form and a radio button to select regarding consent. If participants consented, they were presented with modules on separate pages assessing: (1) demographic characteristics; (2) prior research experience; (3) mobility (ie, use of aids; driving frequency); (4) tech ownership (ie, own a tablet, smartphone, home Wi-Fi); (5) social media usage (ie, frequency of using Facebook, Twitter, Snapchat, Instagram, TikTok, WhatsApp); (6) willingness and extent of contributing various data (ie, across categories of activities, sensors, metadata); (7) opinions about data controls; and (8) willingness to use a robot for Instrumental Activities of Daily Living (IADLs). This survey totaled 52 questions over 12 pages presented in the same order for all participants (see Multimedia Appendix 1 for full survey). Questions for demographic reporting required responses, while all other items only suggested responses. Participants were only able to navigate forward and not backward through the survey.

### Data Preparation and Analysis Plan

This study reports findings related to the willingness and extent of contributing various data and opinions about data controls. All data were processed using R software (version 4.0.2; R Foundation for Statistical Computing) [9], using the *tidyverse* package [10], including *ggplot2* for data visualization [11] and *dplyr* for data wrangling [12].

## Results

### Sample Characteristics

The sample was 44% female (n=652), with a mean age of 36 (SD 11.97, 20-84). A total of 99% (n=1479) of the individuals owned a mobile phone and 98% (n=1464) had access to home Wi-Fi (Table 1).

**Table 1.** Sample characteristics from online study.

Characteristics	Participants (N=1489)
Age (years), mean (SD; range)	36.5 (12.0; 20-84)
Age ≥50, years, n (%)	224 (15.04)
Past research experience, n (%)	1238 (83.14)
Sex, n (%)	
Male	811 (54.4)
Female	652 (43.7)
Other	19 (1.27)
Proportion Unspecified + No Report	4 (0.2)
Race/Ethnicity, n (%)	
Hispanic	116 (7.8)
White	1134 (76.15)
Black	156 (10.5)
Asian	193 (12.9)
American Indian, Alaska Native	22 (1.5)
Other	27 (1.8)
Average miles driven per week, mean (SD)	78 (181.9)
Median miles driven per week, IQR	29 (5-80)
Technology ownership, n (%)	
Tablet	929 (62.39)
Smartphone	1479 (99.3)
Activity tracker	660 (44.3)
Home Wi-Fi	1464 (98.3)
Home Assistant (eg, Alexa)	812 (54.5)
Awareness regarding Google Takeout	217 (14.6)

### RQ1: What Data Are People Willing to Contribute?

#### Overview

**Figure 2.**
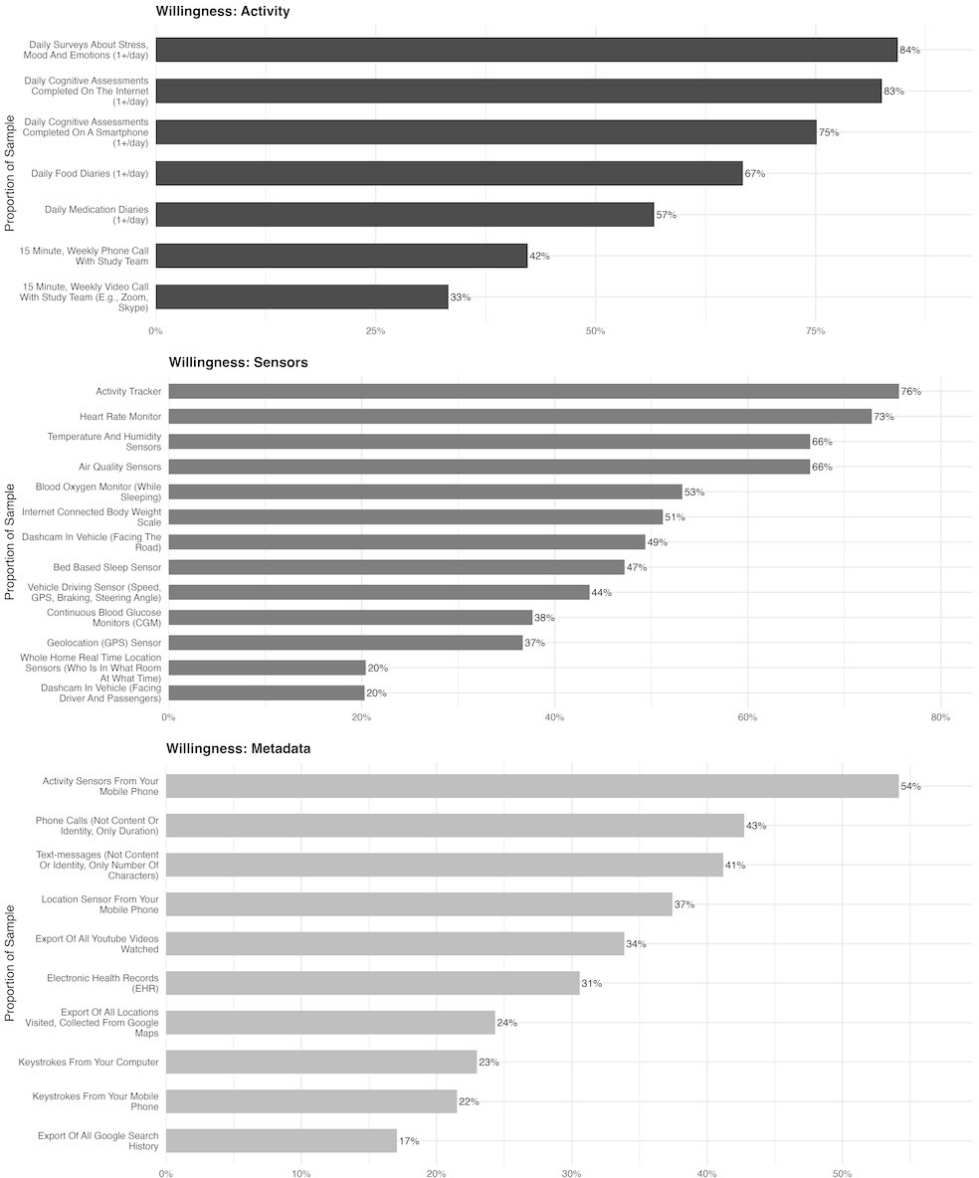
(A) Respondents’ willingness to complete various activities in the context of research; (B) Respondents’ willingness to wear various sensors in the context of research; (C) Respondents’ willingness to share various metadata in the context of research.

For each category of data, the sections below report the most and least endorsed options, as summarized in Figure 2.

#### Activities

Among the activities assessed that could potentially take place in the context of research, 15-minute video or phone calls were the least endorsed options. Respondents were most willing to complete daily surveys about stress, mood, and emotions, as well as daily cognitive assessments.

#### Sensors

Of the sensors assessed that could be worn in the context of research, whole home real-time location sensors and in-cabin, driver-facing dashcams were the three least endorsed options. In contrast, the respondents were most willing to wear an activity tracker, a heart rate monitor, and air quality sensors.

#### Metadata

Of the metadata assessed that may be requested in the context of research, a Google Search history export as well as keystroke data (from computer or mobile phone) were the least endorsed. In contrast, the respondents were most willing to share data from smartphone activity trackers as well as phone and SMS metadata (number and duration of calls).

### RQ2: For How Long Are People Willing to Contribute Critical Data Streams?

Participants were asked to consider a hypothetical study in which they were asked to complete three activities (queried independently), and rate how long in days they would be willing to complete each activity, for a maximum of 100 days (see Textbox 1 for full questions): (1) daily assessments of cognition; (2) wear and charge an air quality or pollution sensor; and (3) wear and charge a GPS location sensor.

Textbox 1. **Questions posed to participants in regard to three data collection activities (ie, cognitive assessments, air quality sensing, GPS location sensing).**

Question

Imagine a study, where you were asked to complete daily assessments of your cognition, via short "“brain games"” or "“mini-video games"”. For how many days might you be willing to complete these assessments (up to 100 days d)?

Imagine a study, where you were asked to wear an air pollution sensor daily, clipping it to a work bag or to your clothing. For how many days might you be willing to wear and charge this device (up to 100 days d)?

Imagine a study, where you were asked to wear a GPS location sensor daily, clipping it to a work bag or to your clothing. For how many days might you be willing to wear and charge this device (up to 100 days d)?

Paired sample *t* tests were conducted to assess the differential willingness to share data from these three critical data streams, each previously implicated in cognitive health research (ie, mobile cognitive assessments, location data (GPS), and ambulatory air quality monitoring). On average, people were willing to complete ambulatory cognitive assessments for 56.7 (SD 36.2) days, air quality monitoring for 58.1 (SD 37.7) days, and GPS location monitoring for 37 (SD 39.0) days. Comparing mobile cognitive assessments and air quality monitoring—participants were willing to complete each for a similar period of time (ie, mean difference of approximately 1.4 d; *t*_1488_=−1.44; *P*=.14). Upon comparing mobile cognitive assessments and GPS location monitoring, participants were willing to complete cognitive assessments for close to 20 more days than GPS location monitoring (ie, mean difference of approximately 19.7 d; *t*_1483_=−17.89; *P*<.001). Comparing mobile air quality monitoring and GPS location monitoring – participants were willing to complete air quality monitoring for close to 21 more days than GPS location monitoring (ie, mean difference of approximately 21 days; *t*_1483_=−20.65; *P*<.001).

### RQ3: What Do People Expect From Their Data?

**Figure 3.**
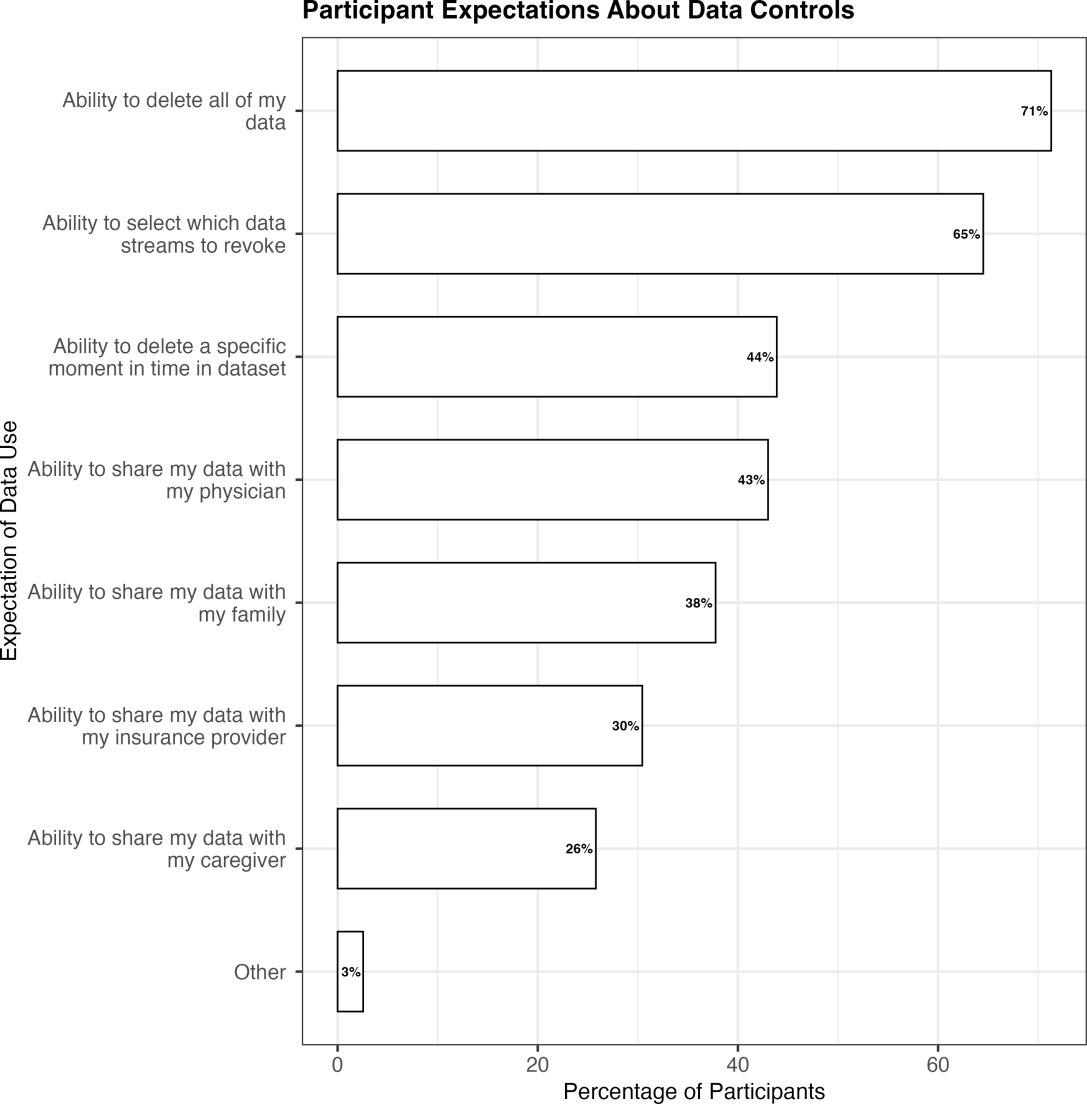
Respondents’ expectations for data controls as it relates to research.

Participants were asked: “Please select the types of control you expect to have over the data submitted as part of a research study” Among the data control and sharing aspects assessed, 1042 (70%) participants were most interested in the ability to delete all their contributed research data, followed by the ability to select specific data streams for deletion (65%, 960) and deleting a specific moment in time (44%, 653). Respondents were least interested in the ability to share data with their insurance providers and caregivers (n=453, 30% and n=384, 25%, respectively; Figure 3).

**Figure 4.**
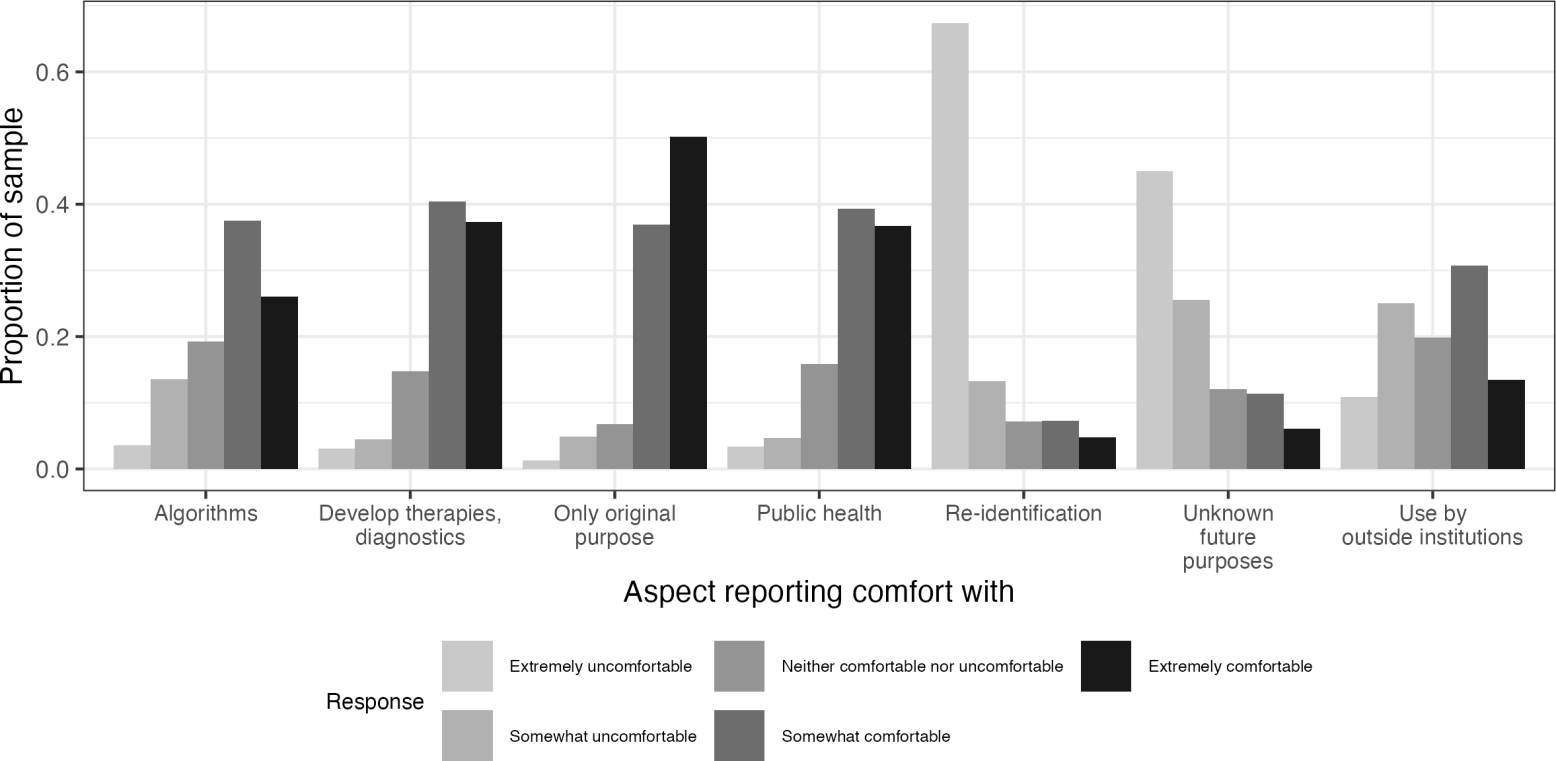
Proportion of sample respondents reporting their comfort levels with various aspects of data usage. The x-axis categorizes different data usage aspects. The y-axis represents the proportion of respondents. Responses range from “Extremely uncomfortable” to “Extremely comfortable,” indicated by different shades of gray.

When asked “Please rate how comfortable (5-point scale, from extremely comfortable to extremely uncomfortable) you would feel about each of the following situations related to your hypothetical data,” participant responses varied by data usage scenario (see Figure 4): (1) data used only for the purpose of the original study; (2) data used to develop algorithms; (3) data used by outside institutions collaborating on this research; (4) data used indefinitely for purposes unknown today; (5) data used to identify the participant; (6) data used to solve a public health problem; (7) data used to develop therapies and diagnoses. Unsurprisingly, participants were mostly uncomfortable with reidentification and with data being used for unspecified future purposes.. Respondents were mixed in regard to data being used by external collaborations. Participants were mostly comfortable with data being used for the original purpose, to develop therapies and diagnoses, and for algorithm development.

## Discussion

### Summary of the Findings

#### RQ1: What Data Are People Willing to Contribute?

Overall, participants were most willing to share data streams that have clear health implications (eg, activity sensor, heart rate monitors, air quality monitors) and least willing to share sensitive data streams (eg, GPS locations, driver/passenger-facing dashcam footage, Google search history). Participants were most willing to share data from active data collection activities and were least willing to share passive data streams (eg, sensors, metadata). People’s willingness to share data that has obvious health implications replicates prior findings in this area [4] and underscores the necessity of education on the relevance of each data stream to health outcomes, if it is to be considered and adopted by research participants.

#### RQ2: How Long Are People Willing to Contribute Critical Data Streams?

On average, participants are willing to share data for up to 37 days, though this varied by data stream assessed. Willingness to share GPS location data was lowest, highlighting participant privacy concerns associated with data that may be more sensitive or identifiable, as was seen with the Strava data leak [6]). In contrast, daily cognitive assessments and ambulatory air quality monitoring, were similarly endorsed, further supporting engagement with data streams with clear health implications.

#### RQ3: What Controls and Feedback Do People Expect From Their Data?

Most participants endorsed the desire to delete all or specific streams of research data contributed by them. Although these data-related protections are required as per IRB regulations, most digital health technologies used for research do not necessarily have a straightforward method using which a person may delete their contributed data. This often requires a direct communication with the research group and a manual backend process. Future iterations of app- and cloud-based digital health technologies may want to consider affording the data provider (ie, the participant) more control over their data, including ways to download, remove data as desired, as well as mechanisms by which they can easily share data with key stakeholders in their life (eg, family or physician). Google Takeout is a platform through which Google account users can export all data originating from Google applications. In this study, only 14% individuals were aware of this tool, highlighting the need for data sharing education. Regarding various data usage scenarios, participants were mostly uncomfortable with unknown data use scenarios and reidentification, and comfortable with data being used to develop therapies and diagnoses, especially for the original purpose. Interestingly, participants reported mixed responses about their ratings of comfort, in regard to data being used by external collaborators. These findings highlight the importance of being transparent with participants on the various ways the data are being used—as not all data use scenarios are seemingly acceptable.

### Limitations and Future Directions

Although a large sample (ie, of approximately 1500 respondents) was recruited for this study, a major limitation is that participant demographics indicate a recruitment bias toward midlife, highly educated (69% have associates degree or higher), White participants (76%, n = 1134), and individuals active on online research platforms. As participation required familiarity with digital interfaces and online survey platforms, the sample may not adequately reflect the views of individuals with low technology adoption rates—particularly those in rural or socioeconomically disadvantaged communities [13]. Future research should consider probing similar research questions, leveraging a representative and more diverse sample of respondents, especially considering past research has shown differential willingness to share various data as a function of education and minority status. The extent to which older adults (ie, >60 years) are aware of and are willing to participate by sharing sensor data and metadata is unclear. Older adults were sparsely represented in this sample, limiting insight into the acceptability of sensor-based monitoring among individuals most likely to benefit from digital health interventions (eg, including those aimed at chronic disease mitigation or cognitive decline). Age-related factors such as cognitive load, sensory impairments, or comfort with technology may interact with perceived burden; however, these nuances were not explored in this study.

In addition, this study did not directly measure aspects of personality, or expectations for data privacy—two factors likely to explain variance in willingness to share data across the spectrum of participant burden. Participants’ decisions may have been influenced by recent media exposure or personal experiences related to data breaches or algorithmic discrimination, but these factors were not assessed. It is expected that awareness of specific events that highlight the reidentification potential of various sensitive data (eg, GPS) may moderate willingness to share sensitive data, along with the personality constructs of openness and consciousness [14]).

### Conclusions

Importantly, the study relies on self-reported, hypothetical willingness to share data, which may not correspond with real-world behavior, particularly when actual study conditions involve real data collection amidst daily life schedules and hassles. Future work should validate these findings in live digital health trials to assess whether stated willingness translates to actual participation and data-sharing compliance. This study aimed to elucidate the types of data and control measures that participants expect in research. These findings can guide the optimization of study protocols to enhance participant engagement, data quality, and minimize participant burden. Ensuring participant privacy is paramount, and researchers have a duty to transparently communicate the rationale for data collection and its potential benefits. As researchers intend to collect rich data across the Active-Passive data continuum (see Figure 1), our participant’s privacy comes first, and it is our duty to inform them why we collect the data we do. This approach will foster trust and encourage greater participation in research involving potentially sensitive data streams.

## Supplementary material

10.2196/64082 Multimedia Appendix 1 Data for the willingness to participate, and share various streams.

## Abbreviations

IRB: institutional review board

## References

[1] Revilla M, Couper MP, Ochoa C (2019). Willingness of online panelists to perform additional tasks. Methods, Data, Analyses: A Journal for Quantitative Methods and Survey Methodology (Mda).

[2] Wenz A, Jäckle A, Couper MP (2019). Willingness to use mobile technologies for data collection in a probability household panel. Surv Res Methods.

[3] Struminskaya B, Lugtig P, Toepoel V, Schouten B, Giesen D, Dolmans R (2021). Sharing data collected with smartphone sensors: willingness, participation, and nonparticipation bias. Public Opin Q.

[4] Seltzer E, Goldshear J, Guntuku SC (2019). Patients’ willingness to share digital health and non-health data for research: a cross-sectional study. BMC Med Inform Decis Mak.

[5] Keusch F, Wenz A, Conrad F (2022). Do you have your smartphone with you? Behavioral barriers for measuring everyday activities with smartphone sensors. Comput Human Behav.

[6] Hsu J The strava heat map shows even militaries can’t keep secrets from social data. Wired.

[7] Hysong SJ, Khan MM, Petersen LA (2011). Passive monitoring versus active assessment of clinical performance: impact on measured quality of care. Med Care.

[8] Quality data from real people for faster breakthroughs. Prolific.

[9] (2021). The R Project for Statistical Computing. The R Foundation.

[10] Wickham H, Averick M, Bryan J (2019). Welcome to the Tidyverse. JOSS.

[11] Wickham H (2011). ggplot2. WIREs Computational Stats.

[12] Wickham H, Francois R, Henry L, Müller K, Dplyr (2014). Dplyr: A Grammar of Data Manipulation. R Package Version 0.8.4.. https://CRAN.R-project.org/package=dplyr.

[13] Pethig F, Kroenung J, Noeltner M (2021). A stigma power perspective on digital government service avoidance. Gov Inf Q.

[14] Linek SB, Fecher B, Friesike S, Hebing M (2017). Data sharing as social dilemma: Influence of the researcher’s personality. PLOS ONE.

[15] Nelsonroque/cascadelab_apdcm_mhealth. GitHub.

