# Supplementary material for: Acceptability of Active and Passive Data Collection Methods for Mobile Health Research: Cross-Sectional Survey of an Online Adult Sample in the United States
Source: JMIR Form Res. 2025 Sep 12;9:e64082. doi: 10.2196/64082 (PMC12431785; doi:10.2196/64082)
Supplement: Multimedia Appendix 1 [file formative-v9-e64082-s001.docx]

**Supplemental Materials**

Willingness to Participate, and Share Various Data Streams (i.e., data underlying Figures 2-5).

| **Metric** | **N** | **Proportion of Sample** | **Type** |
| --- | --- | --- | --- |
| 15 minute weekly phone call with study team | 630 | 0.42 | Activities |
| 15 minute weekly video call with study team e g zoom skype | 496 | 0.33 | Activities |
| daily cognitive assessments completed on a smartphone 1 or more per day | 1119 | 0.74 | Activities |
| daily cognitive assessments completed on the internet 1 or more per day | 1229 | 0.81 | Activities |
| daily food diaries 1 or more per day | 994 | 0.66 | Activities |
| daily medication diaries 1 or more per day | 844 | 0.56 | Activities |
| daily surveys about stress mood and emotions 1 or more per day | 1256 | 0.83 | Activities |
| activity tracker | 1127 | 0.75 | Sensors |
| air quality sensors | 990 | 0.66 | Sensors |
| bed based sleep sensor | 704 | 0.47 | Sensors |
| blood oxygen monitor while sleeping | 793 | 0.53 | Sensors |
| continuous blood glucose monitors cgm | 561 | 0.37 | Sensors |
| dashcam in vehicle facing driver and passengers | 302 | 0.20 | Sensors |
| dashcam in vehicle facing the road | 735 | 0.49 | Sensors |
| geolocation gps sensor | 547 | 0.36 | Sensors |
| heart rate monitor | 1085 | 0.72 | Sensors |
| internet connected body weight scale | 762 | 0.50 | Sensors |
| temperature and humidity sensors | 990 | 0.66 | Sensors |
| vehicle driving sensor speed gps braking steering angle | 649 | 0.43 | Sensors |
| whole home real time location sensors who is in what room at what time | 305 | 0.20 | Sensors |
| activity sensors from your mobile phone | 807 | 0.53 | Metadata |
| electronic health records ehr | 455 | 0.30 | Metadata |
| export of all google search history | 254 | 0.17 | Metadata |
| export of all locations visited collected from google maps | 362 | 0.24 | Metadata |
| export of all you tube videos watched | 504 | 0.33 | Metadata |
| keystrokes from your computer | 342 | 0.23 | Metadata |
| keystrokes from your mobile phone | 320 | 0.21 | Metadata |
| location sensor from your mobile phone | 557 | 0.37 | Metadata |
| phone calls not content or identity only duration | 636 | 0.42 | Metadata |
| text messages not content or identity only number of characters | 613 | 0.41 | Metadata |
| datacontrol ability to delete a specific moment in time in dataset | 653 | 0.43 | Data Controls |
| datacontrol ability to delete all of my data | 1062 | 0.70 | Data Controls |
| datacontrol ability to select which data streams to revoke | 960 | 0.64 | Data Controls |
| datacontrol ability to share my data with my caregiver | 384 | 0.25 | Data Controls |
| datacontrol ability to share my data with my family | 562 | 0.37 | Data Controls |
| datacontrol ability to share my data with my insurance provider | 454 | 0.30 | Data Controls |
| datacontrol ability to share my data with my physician | 641 | 0.42 | Data Controls |
| datacontrol other | 38 | 0.03 | Data Controls |
